# Implementing screening for hypertension in archetypal HIV primary care: a mixed-methods assessment

**DOI:** 10.1186/s12913-022-08362-y

**Published:** 2022-08-15

**Authors:** Lydia Buzaalirwa, Lydia Nambala, Grace Banturaki, Penninah Iutung Amor, Anne Katahoire, Elvin Geng, Aggrey Semeere

**Affiliations:** 1 AIDS Health Care Foundation, Kampala, Uganda; 2 Infectious Diseases Institute, Makerere University College of Health Sciences, Kampala, Uganda; 3 Makerere University College of Health Sciences, Kampala, Uganda; 4 Washington University St. Louis, St. Louis, MO USA

**Keywords:** Hypertension screening, HIV-infection, Sub-Saharan Africa, East Africa, Uganda, HIV primary care, Implementation

## Abstract

**Background:**

High prevalence of HIV and hypertension in sub-Saharan Africa puts adults living with HIV (ALWH) at high risk of end-organ complications. Both World Health Organization (WHO) and national guidelines recommend screening and treatment of hypertension among ALWH on antiretroviral therapy (ART). We evaluated the implementation of hypertension screening among adults on ART at three Uganda Cares Primary care facilities.

**Methods:**

Using a sequential explanatory mixed-methods approach, we reviewed patient records, and interviewed both patients and providers during 2018 and 2019. We obtained demographics, clinical and blood pressure (BP) measurements via records review. We estimate the period prevalence of screening and use adjusted modified Poisson regression models to evaluate predictors of screening. In-depth interviews were analysed using a thematic approach to explain the observed prevalence and predictors of BP screening.

**Results:**

Records for 1426 ALWH were reviewed. Patients had a median age of 35 years and 65% of them were female. Most were on ART (89% on first-line) with a median duration of 4 years. Only 262 (18%) were overweight or obese with a body mass index (BMI) > 25 Kg/M^2^. In 2017 or 2018 patients made a median of 3 visits and 783 patients had a BP recorded, hence a period prevalence 55%. Older age, male sex, more clinic visits, and clinic site were associated with screening in the adjusted analyses. Erratic BP screening was corroborated by patients’ and providers’ interviews. Challenges included; high patient numbers, low staffing, provider apathy, no access to treatment, and lack of functioning of BP equipment.

**Conclusion:**

Almost half of regular HIV clinic attendees at these prototypical primary care HIV clinics were not screened for hypertension for a whole year. Improving BP screening requires attention to address modifiable challenges and ensure local buy-in beyond just providing equipment.

## Background

The high population prevalence of hypertension in sub-Saharan Africa means about 26 million adults living with HIV (ALWH) are at risk for both hypertension and its related complications [1]. Increasing access to Antiretroviral therapy (ART) for ALWH in sub-Saharan Africa has reduced the HIV/AIDS related morbidity and mortality but is likely to lead to increased burden of hypertension given better survival [2]. Compared to the HIV-uninfected, studies suggest that ALWH and hypertension have a 40 to 70% higher risk for end organ complications [3–7]. Uniquely, hypertension is a major potentially modifiable risk factor for these complications. Therefore hypertension ought to be diagnosed, managed and controlled to lessen the untoward burden and possible death from the complications [8–12].

Diagnosing hypertension requires routine blood pressure (BP) measurement, a key first step that typically happens during primary care [13–15]. Since most ALWH regularly attend HIV primary care clinics, these visits offer a unique opportunity for BP screening. Upon diagnosis, management is then initiated and it mainly includes lifestyle modification and medications. If optimized, these interventions are sufficient to achieve BP control and are within the reach of most patients to prevent related complications [16–19]. Recent World Health Organization (WHO) [17, 20, 21] and Uganda National Antiretroviral therapy (ART) treatment guidelines [18], recommend screening and management of hypertension to further enhance longevity, and quality of life for ALWH. Recommendations are based on evidence that ALWH with hypertension are at higher risk of both cardio and cerebrovascular disease compared to HIV-uninfected counterparts [3–7, 22]. Notably, increasing age [23, 24]; impact of inflammatory processes (HIV viral replication and opportunistic infections) [25, 26], and also cumulative effects of ART use [27–29] could contribute to this risk. Effective screening programs therefore are needed within HIV primary care to identify and treat patients with hypertension to prevent related complications. Various models for integrating BP screening into HIV care have been suggested [30]. Proposals include: combination with voluntary HIV counselling and testing [31], during HIV primary care visits [32, 33], and via community differentiated care delivery [32]. Rationale and effectiveness of these approaches are yet to be fully evaluated [34]. With millions of ALWH accessing ART mostly through primary care, it is unclear how well BP screening recommendations have been implemented given the several integration approaches and care models available.

We performed an explanatory sequential mixed-methods study to evaluate BP screening among ALWH on ART and explored patients’ perceptions and providers’ practices regarding hypertension screening at HIV primary care clinics run by AIDS Healthcare Foundation (AHF) Uganda Cares.

## Methods

### Overall design

Using an explanatory sequential mixed-methods approach, we first performed a cross sectional study to estimate period prevalence of hypertension screening and its determinants, followed by in-depth interviews to explain the screening experience at three HIV primary care facilities in Uganda run by Uganda Cares. Participating clinics included; the urban St. Balikudembe market clinic, in Kampala city; semi-urban clinic at Masaka regional referral hospital in south central Uganda, and Kalisizo district hospital clinic, a rural hospital further south towards the Tanzania border. Between March 2018 and March 2019, we reviewed records for ALWH on ART seen during the previous calendar year. Visits were assessed for BP measurements and/or hypertension diagnosis. We then performed in-depth interviews after quantitative analysis on a random sample of the patients and clinic providers to corroborate BP screening information learned.

### Study population

**Fig. 1:**
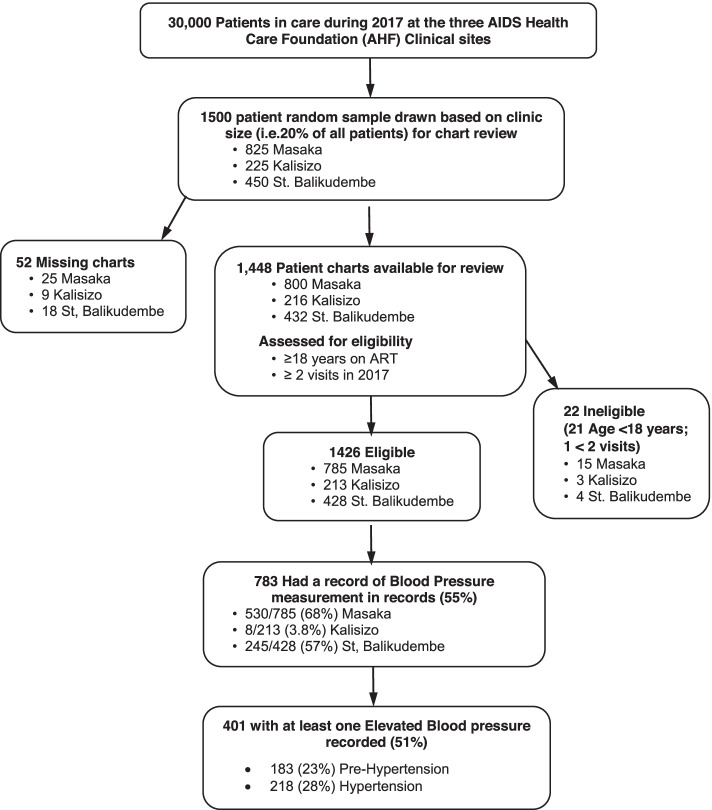
A flow diagram summarizing sampling from the HIV clinics and enrollment into the study

We studied ALWH (> 18 years) on ART with at least 2 clinic visits in the previous calendar year. Out of 30,000 eligible patients, we drew a sample of 1500 based on proportionate contribution of all eligible, by clinic site (Fig. 1). During visits in 2017, we identified 825 records from Masaka and 225 from Kalisizo, then in 2018, 450 from St. Balikudembe clinic. For the in-depth interviews we interviewed a random sample of 30 ALWH on ART with at least 2 clinic visits and 20 providers (doctors, nurses, clinical officers, clinic administrators) selected by proportional representation, who had been employed and had worked at the clinic for at least a year in the study period.

### Study sites

Uganda Cares is a non-profit with over 16 years’ experience, providing free HIV prevention, care and treatment services, as well as advocacy. Currently it operates and supports clinics in 23 districts within Uganda and with over 115,000 patients as of July 2021. Uganda Cares’ HIV care program is intent on aligning care delivery with ambient guideline recommendations. Typically, clinics are staffed by clinicians mostly trained and experienced in providing HIV related care and treatment. At the time of this evaluation, BP machines and training to support BP measurement had been given to five Uganda Cares clinics, three of which were studied. We chose the St. Balikudembe, Masaka, and Kalisizo, clinics to represent the urban, semi urban and rural service areas respectively.

### Patient record review

Using patient clinic identification numbers from the sample drawn, charts were identified and reviewed for evidence of BP screening on all visits in the previous calendar year. Specifically, data retrieved included; visit dates, demographic information, HIV treatment information, evidence of screening (BP measurements), hypertension diagnosis and/or record of treatment (prescription and or medication use). Data were abstracted on to a standardized questionnaire and later entered into a REDCap™ database via android tablet.

### In-depth interviews

Upon initial analysis of BP screening prevalence and predictors, we explored patients’ perceptions and providers’ practices based on the findings. A trained non clinical research assistant interviewed both patients and providers. A subset of patients were purposefully sampled based on whether they had been screened or not screened to understand the screening experience. Likewise, providers were purposefully sampled based on their roles in the clinic. During 20 to 30 minute interviews, interviewees responded to open-ended questions regarding how they had observed the implementation of BP screening during routine care at the respective clinic. Digital audio recordings of the interviews were transcribed into the language the interviews were conducted. During transcription all personal identifiers were removed. Audio recordings, transcriptions, notes, and other related records were secured, only accessible by authorized persons.

### Quantitative analysis

We summarised demographic data using descriptive statistics and estimated the period prevalence of screening with 95% confidence intervals as number screened for hypertension out of all in care with two or more visits within the calendar year. We defined screening for hypertension as having at least one record of BP screening within the one-year period [35]. Using modified Poisson regression with robust variance, we evaluated predictors of BP screening and generated prevalence ratios (PR) adjusted for sex, age, clinic site, duration on ART, duration in care, clinic visits, and ART regimen. All analyses used STATA® software version 16.1.

### Qualitative analysis

We used a thematic approach to analyze the interviews. We read through the transcripts to familiarize ourselves with the data, then used deductive coding and analysis, whereby the codes and themes were derived more from concepts and ideas that were embedded in the tools used for data collection. De-identified transcripts were uploaded into ATLAS® ti V8 software for coding. Once the coding process was completed, code reports were generated. Based on these code reports themes were identified, reviewed and refined.

## Results

### Quantitative findings

Out of the intended 1500, we identified 1448 charts and 52 were missing (Fig. 1). Of those identified, 1426 were eligible for review while 22 were ineligible for various reasons (Fig. 1). Majority of the charts were from Masaka (55%), a third from St. Balikudembe (30%) and the rest from Kalisizo (15%) (Table 1). Patients’ median age was 35 years (Interquartile range (IQR): 29 to 43) and two-thirds were women (65%). Patients had been on ART for a median of 4 years (IQR: 2 to 6) with a median duration in HIV care of 4 years (IQR: 1 to 7). Majority (1269, (89%)) were still on a first-line ART with a few on second line regimens. In the previous year, patients made a median of 3 visits (IQR: 2 to 6) to the clinic. From data available, 262 (18%) patients were overweight or obese (body mass index (BMI) > 25 Kg/M^2^).

**Table 1:** Characteristics of adults living with HIV on antiretroviral therapy with clinic visits in 2017 or 2018 whose records were reviewed for evidence of blood pressure screening at three AIDS Health Care Foundation (AHF) supported primary care facilities in Uganda

Characteristic	***N*** = 1426
**Age, years**^**a**^	35 (29-43)
**Male Sex**	35%
**Duration on ART, years**^**a**^	4 (2-7)
**Duration in Care, years**^**a**^	4 (2-7)
**Second line ART use**^**a**^	11%
**Clinic Location**	
Masaka	55%
St. Balikudembe	30%
Kalisizo	15%
**Body Mass Index (BMI) category**^**b**^**, Kg/M**^**2**^	
< 19	28%
19-24	54%
25-30	15%
≥ 30	3.4%
**Number of clinic visits per patient in 2017 or 2018**^**a**^	2 (2-3)

^a^Median (Interquartile Range)

^b^Missing for 51% of patient records

### Period prevalence of screening

Among 1426 patients, we observed a total of 3138 visits with a BP screening. The median number of visits per patient with a BP screened were 2 (IQR: 2 to 4). Overall 783 patients had at least one BP screening recorded at a clinic visit in 2017 or 2018. This represents a period prevalence of 55% (95% CI: 52 to 57%) (Table 2). Screening prevalence was highest in Masaka 67% (95 CI: 64 to 71%) and lowest in Kalisizo 4% (95% CI: 1.9 to 7.4%) (Table 2). Older adults (> 55 years) had the highest screening prevalence of 55% (95% CI: 46 to 67%) compared to other age groups. Among the 783 patients with at least one BP measurement, 183 patients had reading in the Stage 1 hypertensive range (130/80 to 139/89 mmHg) according to the New ACC/AHA High BP guidelines definition [35] corresponding to a period prevalence of 23% (95% CI; 21 to 26) (Table 2). Another 218 patients (28% (95% CI; 25 to 31)), had readings in the Stage 2 hypertensive range (> 140/90 mmHg).

**Table 2:** Period prevalence of blood pressure screening, and grade of hypertension among 1426 adults living with HIV during 2017 or 2018 seen at any one of three HIV primary care facilities in Uganda

	Screening ***N*** = 1426	Stage 1 Hypertension ***N*** = 783	Stage 2 Hypertension ***N*** = 783
	Prevalence (95% CI)	Prevalence (95% CI)	Prevalence (95% CI)
**No. Identified**	783	183	218
**Prevalence**	55% (52 to 57)	23% (20 to 26)	28% (25 to 31)
**Sites**			
*Masaka*	67% (64 to 71)	22% (19 to 26)	30% (26 to 34)
*Kalisizo*	4% (1.9 to 7.4)	13% (1.7 to 54)	63% (24 to 87)
*St. Balikudembe*	57% (52 to 62)	26% (21 to 32)	23% (18 to 29)
**Age group (Years)**			
*18-35*	54% (50 to 58)	23% (19 to 28)	16% (13 to 20)
*36-55*	55% (52 to 59)	24% (20 to 29)	35% (30 to 40)
*> 55*	57% (46 to 67)	15% (7.0 to 28)	60% (45 to 72)

### Determinants of hypertension screening

Adjusting for age, clinic site, duration on ART, duration in care, clinic visits and ART regimen via modified Poisson regression; compared to women, men were less likely to be screened with a PR of 0.85 (95% CI: 0.78 to 0.94; *p* = 0.001) (Table 3). Also, for every 10-year increase in age, the prevalence of screening increased 1.07 fold (95% CI:1.03 to 1.13; *p* = 0.001) after adjusting for other variables (Table 3). Further, adjusted analysis suggested that patients were more likely to get screened if they attended more clinic visits since every 5 clinic visits attended increased likelihood of screening 1.84 fold (95% CI: 1.65 to 2.05); *p* < 0.001) (Table 3). Screening prevalence also depended on clinical care site. Compared to Masaka, patients in Kalisizo (PR 0.06 (95% CI: 0.03 to 0.1; *p* < 0.001)), and St Balikudembe (PR 0.85 (95%CI: 0.77, 0.93; *p* < 0.001)) were less likely to be screened after adjusting for other variables (Table 3). Further we sought to explore some interactions especially clinic visits among men; regimen and clinic visits; age and clinic visits; duration in care and clinic visits. Among males we found a 1.3-fold increase (95% CI:1.1 to 1.6 *p* = 0.015) in probability of screening with more clinic visits but this was 1.15-fold (95% CI: 0.9 to1.4 *p* = 0.163) after adjusting for age, clinic site, duration on ART, duration in care, and ART regimen. The other interactions were unremarkable.

**Table 3:** Adjusted and unadjusted Prevalence Ratios (PR) for blood pressure screening during a clinic visit between 2017 or 2018 among adults living with HIV on antiretroviral therapy at three HIV primary care facilities in Uganda

Characteristic	Unadjusted		Adjusted	
	PR (95%CI)	***P***-Value	^**a**^PR (95%CI)	***P***-Value
**Male, sex**	0.88 (0.70 to 1.00)	0.02	0.85 (0.78 to 0.94)	0.001
**Age, per 10-Year increase.**	1.05 (1.00 to 1.10)	0.04	1.07 (1.03 to 1.13)	0.001
**Duration on ART**	0.99 (0.98 to 1.00)	0.3	0.99 (0.97 to 1.02)	0.7
**Duration in Care**	0.99 (0.98 to 1.01)	0.5	1.01 (0.98 to 1.03)	0.5
**Clinic visits, per 5 visits**	0.82 (0.74 to 0.91)	< 0.001	1.84 (1.65 to 2.05)	< 0.001
**Clinical site**				
*Masaka*	Ref		Ref	
*Kalisizo*	0. 06 (0.03 to 0.10)	< 0.001	0.03 (0.02 to 0.07)	< 0.001
*St. Balikudembe*	0.85 (0.77 to 0.93)	0.001	0.70 (0.63 to 0.78)	< 0.001
**Second line ART**	1.20 (1.01 to 1.32)	0.03	0.92 (0.81 to 1.05)	0.2

^a^Adjusted for sex, age, clinic site, duration on ART, duration in care, Clinic visits made, and ART regimen using modified Poisson regression with robust variance

### Qualitative findings

We performed 50 in-depth interviews among 33 patients and 17 health care workers (Table 4) across all sites. Of these, 21 were conducted at Masaka, 19 at St. Balikudembe and 10 in Kalisizo. Overall females constituted 69% (23) of the patients and 65% (11) of the providers.

**Table 4:** Participants for the in-depth interviews regarding blood pressure screening measurements at three HIV primary care facilities in Uganda

Site	Patients	Health care workers			Overall
		Nurses	Medical Officers	^a^Other	
Masaka	14	3	2	2	21
Kalisizo	6	1	2	1	10
St. Balikudembe	13	2	1	3	19

^a^Includes clinical officers, dispensers and counsellors

### Patients’ perception of screening practice

Generally, patients reported inconsistent screening for hypertension as demonstrated by these quotes:

*“…regarding hypertension whenever I come to the clinic, I am not screened for hypertension… but there is a season when all patients are screened for hypertension”* ***(PM012).***

*“They are inconsistent, sometimes you come and they check but sometimes they don’t check.”* ***(PSB008).***

Screening likely depended on various influences, such as previously diagnosed hypertension as illustrated here:

*“I have had hypertension for 13 years. Whenever I come to the clinic my blood pressure is measured. Sometimes when I come to the clinic I am not screened for hypertension but most of the times we are screened****.” (PM012).***

At St. Balikudembe clinic, another noted that screening has changed overtime with reduced frequency more recently.

*“…They were checking sometime back but they have not been checking me these days”* ***(PSB005).***

While in Kalisizo another reported that screening only started recently.

*“They have just started screening for hypertension when you visit the clinic… Sometimes they screen for blood pressure”* ***(PK005).***

Screening seemed more likely when patients came to clinic earlier in the day.

*“Most of the time when I come to the clinic I must be screened for hypertension; this is why I come early so that I can be screened.” (****PM008).***

Remarkably, upon screening, patients reported insufficient provider communication regarding findings. Some patients perceived the lack of communication, in some instances, as an indicator of normal BP status:

*“They never told me. After screening he just told me move to this next point.”* ***(PM003).***

*“If they have not told me anything, it means I don’t have [high blood] pressure.”* ***(PSB006).***

### Patient’s perceived benefits of screening

Patients reported that routine screening is not only informative of one’s health status but is also the gateway to hypertension treatment.

*“It is good [to screen] because when you know your health condition, you are better than a person who does not know”* ***(PSB003).***

Absence of anti-hypertensive medication at the HIV clinics, and medication cost were also noted as potential impediments to deriving full benefits of screening.

*“…It would be better to get all the medication from this clinic also...”* ***(PM008).***

### Providers’ perspectives on screening

Most providers recognized the importance of screening for hypertension among ALWH on ART.

*“…We don’t have the statistics here but based on my own experience …. I think out of 10 patients I see in a day, 3 of them are hypertensive****.” (HWSB002).***

They reported however that screening was not necessarily emphasized across clinic facilities. For instance, the Masaka clinic allocated a day per week to screen older adults for hypertension among other issues.

*“Right now, we are seeing many cases among the elderly. That’s why we have decided to allocate a day in the week on Wednesday which is for seniors… so that they don’t miss those routine services like BP, RBS [Random Blood Sugar] …”* ***(HWM004).***

Providers stated some challenges that imped regular screening, among them: the high patient numbers, limited staff and, few and/or defective BP machines. Providers stated:

*“Some patients are not screened because we are busy, [and] we have to change, sometimes we divide ourselves.”* ***(HWK004).***

*“…But then there are days that are actually very heavy [with many clients] and basing on the staffing, it makes it hard [to] screen everyone.”* ***(HWK001).***

*“The challenge is once in a while, the [B.P] machine is down and the nurse is over whelmed so they say no… By the time we get the cells [batteries], more than 20 patients have gone [without screening]”* ***(HWSB002).***

Providers’ indifference to screening was also stated as a reason for inconsistent screening.

*“I know what the ideal is, only that sometimes it is not done due to some laxity…sometimes they screen then next week they don’t.”* ***(HWK002).***

*“Some health staff, feel like rushing clients and so they miss taking their blood pressure”* ***(HWM004).***

Providers also recounted that screening without access to anti-hypertensive medicines is a big challenge, suggesting that even just providing basic or some of the BP drugs would be a good start.

*“…but the biggest challenge is we lack the essential hypertensive drugs… I think if you can give someone nifedipine they can buy the rest a few drugs not all drugs”* ***(HWSB002).***

Notably, BP measurements were documented for action by clinicians, even when the patient was not meant to see a clinician.

*“…For those who go through the “fast track”, we just write their [ART] drugs in the dispensing sheet. …we record the weight and the BP such that if there is anything wrong then that patient immediately goes back to the clinician”* ***(HWM004).***

We observed a lower likelihood of screening among men. Providers reported that most men requested many months’ worth of ART hence made fewer clinic visits.

*“… for men; they may ask for more than three months of drugs due to the nature of their work… but for women, if you tell them I want to see you after one month, they have no problem with that.”* ***(HWM004).***

## Discussion

Screening by routine BP measurement is a critical initial step in identifying undiagnosed or uncontrolled hypertension. It provides the opportunity to initiate and optimize management  of high BP especially among high risk groups such as ALWH. Primary HIV care provides an ideal setting for screening since patients are required to routinely attend care [31, 32]. Using a mixed-methods approach, we evaluated implementation of BP screening within routine HIV care at three archetypal clinics run by Uganda Cares. First we estimated the period prevalence of screening and its determinants, then explored patients’ and providers’ perceptions to clarify observed screening prevalence and determinants.

Among these ALWH on ART, just over a half of them (55%) were screened for hypertension at a clinic visit made in the prior year (2017 or 2018). This screening frequency was higher than  findings from a clinical trial among ALWH in Eastern Uganda [36],  which reported a lower (28%) prevalence of screening between 2014 and 2017 [37]. We also observed wide variation in BP screening prevalence between the three facilities in our study ((4 to 67%), Table 2). This dissimilarity between studies and within our study sites just illustrates the erratic implementation and variation in practice within HIV care facilities in the region. Disparity in screening practice is supported by the interview findings from patients who stated screening being done haphazardly across and within clinic sites. Providers described several unique challenges that could in part explain this variability and we discuss them below. Overall, screening was suboptimal despite patients’ regular clinic attendance representing a squandered opportunity to identify either undiagnosed or uncontrolled hypertension in a high risk population. Remarkably about a third (28%) of measurements revealed non-ignorable elevated BPs requiring attention either as new diagnoses or uncontrolled BP. We had insufficient data to delineate between new diagnoses nor uncontrolled BP. That said, realising benefits from screening requires systematic investment in approaches to enhance and routinize consistent implementation.

Male sex, age, more frequent clinic visits and clinical site were statistically significant predictors of screening in adjusted models (Table 3). Previous studies have documented predictors for hypertension, but not for its screening among ALWH [38]. Therefore, interviews with providers and patients were critical in clarifying these observations. We established that men were less likely to get screened. In the general population, studies suggest that men, compared to women, tend to be less aware of their hypertension status [39, 40]. From our interviews, the nature of work which typically takes most men away from home for long periods of time was suggested as a reason for infrequent visits and hence lower BP screening probability. Providers suggested that men also request ART for many months, hence avoiding clinic visits. This reasoning could in part be supported by our interaction analysis result which suggested higher probability of screening among men with more visits, though not statistically significant in the adjusted analysis. Previously, less frequent engagement of males in HIV care services has been reported but not specifically in relation to hypertension screening [41–43].

Increasing age was more likely associated with higher probability of screening. The Masaka clinic had a weekly special clinic day for older adults and this provided an opportunity to prioritize relevant issues like BP screening. Besides, we learnt that those previously diagnosed (especially older adults) with hypertension, are more likely to be screened either because patients request or clinicians managing patients with hypertension take BP measurements prior to prescribing medicines. We couldn’t empirically check this since previous diagnoses were unavailable. Given that risk of hypertension increases with age, and older adults interface with health services more frequently, they are therefore more likely to be screened and hence made aware of their hypertension status [39, 44].

Moreover, frequent visits predicted screening because higher clinic visit frequency increases propensity of being screened, even for non-clinician visits. Over the last 10 years, HIV care programs have initiated Differentiated Service Delivery (DSD) models for sustainable HIV care delivery [30]. Most of these models require patients to make fewer clinic visits or spend less time at clinics reducing opportunities for BP screening. Since 2012, Uganda Cares has provided ART via fast-track and multi-month dispensing DSD approaches hence potentially reducing opportunities of clinician interaction for BP screening. Such DSD HIV care implementation should therefore figure out how to accommodate for hypertension screening even with limited clinician interaction.

**Fig. 2:**
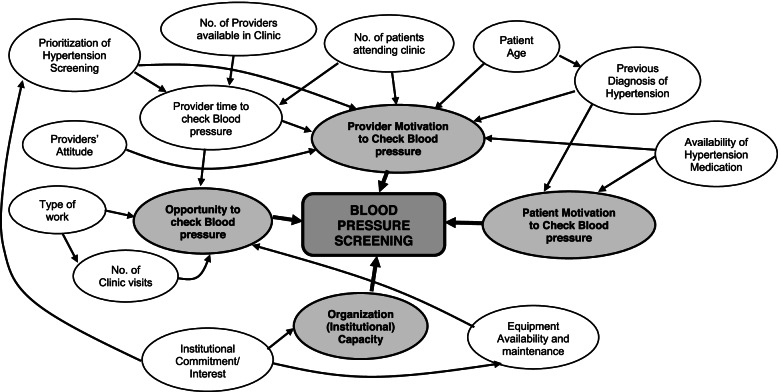
A casual logic model summarizing the relationship between the factors influencing blood pressure screening at the study facilities. From our qualitative and quantitative findings, we hypothesize that the key influences of blood pressure screening operate mainly via: provider and patient motivation, opportunity to measure blood pressure and the organization (institutional) capacity

Patients at the Masaka clinic were more likely to be screened compared to the other two sites. Site level differences gleaned from the interviews suggest that administrative (institutional) importance attached to screening could explain this difference in performance. While for instance a dedicated clinic was established weekly to attend to health issues for older adults in Masaka (including hypertension), other sites had none. Also large patient numbers with few clinicians, could have contributed to inconsistent screening since patients and providers noted that measurement varied with the time of day and how busy the clinic was. This has previously been suggested as a challenge [14, 31]. Despite the numbers and congestion, some clinics did manage to screen patients regularly by prioritizing them on given days. Similar approaches prioritizing high risk patients such as older adults, pregnant women and even men, could be a considered solution. Importantly, all three facilities had similar equipment and support from Uganda Cares to implement screening, but we found varying performance. This suggests a need to identify and address unique local administrative challenges to ensure optimal implementation. The interplay of these and other determinants with regard to BP screening are summarised causal logic model (Fig. 2).

Both patients and providers mentioned the need for BP screening with access to treatment. BP screening with access to antihypertensive treatment has heretofore been suggested for ALWH [32, 33]. Indeed, screening could be unavailing if patients cannot appropriately access therapy. Presently, patients screened and diagnosed or assessed with uncontrolled BP in HIV primary care either privately purchase, or attempt to obtain free medicines at government facilities. Besides requiring another clinic visit on a different day, government facilities are susceptible to stock-outs of medications. Patients therefore opt to purchase medicines out-of-pocket which is unsustainable for most due to cost [45]. Inaccessible treatment is likely a disincentive for consistent screening by both providers and patients. A robust hypertension screening program in HIV primary care requires linkage to sustainable treatment.

To summarise and aid conceptualization of the complex interrelationships between various factors we found to explain the low screening prevalence, we provide a casual logic model (Fig. 2). Our intention is to provide a framework to facilitate targeted interventions to enhance screening for hypertension in the region. From what we found, the suggested factors could be considered to contribute to either: provider and patient motivation, opportunity in the clinic, or the organizational (institutional) capacity to screen for BP. While there are several possible interrelationships between the factors, our findings suggest that most can be thought to influence screening via these four domains. Therefore, we propose that interventions to enhance screening in a similar clinical setting should at a minimum target these domains in a bid to enhance screening for hypertension.

Our study did have some limitations. First, while Uganda Cares provides ideal HIV care, our findings might not fully represent all HIV care facilities in Uganda or the region. Nonetheless, the clinics represented here on average epitomize what could happen to analogous HIV clinics. Secondly, we relied on clinic records for evidence of hypertension screening and could have missed other screening performed and not recorded. Interviews with providers informed us that overall, records are kept well and hence at best represent practice at the facilities. Also data integrity is part of the institutional culture at Uganda Cares since the Masaka clinic is a participating site for International Epidemiologic Databases to Evaluate AIDS consortium [46]. This means our estimated prevalence of screening could be an underestimate but still way below the ideal 100%, given that patients’ and provider’s alike consistently corroborated suboptimal performance. We didn’t have data to confirm previous or ongoing hypertension diagnoses and treatment status for participants, and as such couldn’t delineate between new diagnoses vs uncontrolled BP among those with higher than normal BP measurements. This was not a primary goal of the study. However, both clinical assessments do rely on the same mechanism of BP screening whose implementation we found suboptimal and we provide areas of focus to improve its execution.

There are several implications of our findings. We observed suboptimal hypertension screening probability averaged over a year which varied widely between sites due both modifiable and non-modifiable reasons. We also identified what could make screening more or less likely. Attention to modifiable challenges specifically: patient numbers, staffing, provider indifference, access to treatment, and availability and functionality of BP machines could improve screening. We provide a framework to guide intervention design and policies to enhance screening (Fig. 2). First is provider and patient motivation which are key in generating demand for screening. Secondly, we ought to increase opportunity for screening. Whatever approach is chosen for HIV and hypertension care integration [32], increasing likelihood of screening seems to require more interaction with providers who can measure BP, even within current DSD models. There are opportunities of enhancing screening via prioritization of special populations. Lastly, local administrative and organisation capacity is critical to institutionalization of screening practice and hence helps perpetuate and sustain implementation.

## Conclusion

We observed sub-optimal BP screening among regular clinic attendees on ART and in routine HIV primary care. Sex, age, clinic visits and clinical site were statistically significant predictors of hypertension screening in adjusted models and this was corroborated by the interviews with patients and providers. Prevalence of screening was inconsistent and varied across clinical sites largely due to modifiable factors that influence provider and patient motivation, opportunity and organisational capacity. These domains provide potential targets for enhancing screening even with evolving HIV care.

## Data Availability

The datasets analysed during the current study are not publicly available due to the governing policies of the AHF-Uganda Cares program but are available from the corresponding author on reasonable request.

## References

[1] Organization WH (2013). A global brief on hypertension : silent killer, global public health crisis: world health day 2013.

[2] Nduka CU, Stranges S, Sarki AM, Kimani PK, Uthman OA (2016). Evidence of increased blood pressure and hypertension risk among people living with HIV on antiretroviral therapy: a systematic review with meta-analysis. J Hum Hypertens.

[3] Chow FC, Regan S, Feske S, Meigs JB, Grinspoon SK, Triant VA (2012). Comparison of ischemic stroke incidence in HIV-infected and non-HIV-infected patients in a US health care system. J Acquir Immune Defic Syndr.

[4] Freiberg MS, Chang CC, Kuller LH, Skanderson M, Lowy E, Kraemer KL, Butt AA, Bidwell Goetz M, Leaf D, Oursler KA, Rimland D, Rodriguez Barradas M, Brown S, Gibert C, McGinnis K, Crothers K, Sico J, Crane H, Warner A, Gottlieb S, Gottdiener J, Tracy RP, Budoff M, Watson C, Armah KA, Doebler D, Bryant K, Justice AC (2013). HIV infection and the risk of acute myocardial infarction. JAMA Intern Med.

[5] Islam FM, Wu J, Jansson J, Wilson DP (2012). Relative risk of cardiovascular disease among people living with HIV: a systematic review and meta-analysis. HIV Med.

[6] Marcus JL, Leyden WA, Chao CR, Chow FC, Horberg MA, Hurley LB, Klein DB, Quesenberry CP, Towner WJ, Silverberg MJ (2014). HIV infection and incidence of ischemic stroke. AIDS..

[7] Paisible AL, Chang CC, So-Armah KA, Butt AA, Leaf DA, Budoff M, Rimland D, Bedimo R, Goetz MB, Rodriguez-Barradas MC, Crane HM, Gibert CL, Brown ST, Tindle HA, Warner AL, Alcorn C, Skanderson M, Justice AC, Freiberg MS (2015). HIV infection, cardiovascular disease risk factor profile, and risk for acute myocardial infarction. J Acquir Immune Defic Syndr.

[8] Peck RN, Shedafa R, Kalluvya S, Downs JA, Todd J, Suthanthiran M, Fitzgerald DW, Kataraihya JB (2014). Hypertension, kidney disease, HIV and antiretroviral therapy among Tanzanian adults: a cross-sectional study. BMC Med.

[9] Semeere AS, Sempa J, Lwanga I, Rosalind P-R, Kambugu A. Hypertension and associated risk factors in individuals infected with HIV on antiretroviral therapy at an urban HIV clinic in Uganda. Lancet Glob Health. 2014;2(Special Issue, S23):23.

[10] Kalyesubula R, Kayongo A, Semitala FC, Muhanguzi A, Katantazi N, Ayers D, Forrest JI, Mills EJ (2016). Trends and level of control of hypertension among adults attending an ambulatory HIV clinic in Kampala, Uganda: a retrospective study. BMJ Glob Health.

[11] Okello S, Kanyesigye M, Muyindike WR, Annex BH, Hunt PW, Haneuse S, Siedner MJ (2015). Incidence and predictors of hypertension in adults with HIV-initiating antiretroviral therapy in South-Western Uganda. J Hypertens.

[12] Sander LD, Newell K, Ssebbowa P, Serwadda D, Quinn TC, Gray RH, Wawer MJ, Mondo G, Reynolds S (2015). Hypertension, cardiovascular risk factors and antihypertensive medication utilisation among HIV-infected individuals in Rakai, Uganda. Tropical Med Int Health.

[13] Rabkin M, Nishtar S (2011). Scaling up chronic care systems: leveraging HIV programs to support noncommunicable disease services. J Acquir Immune Defic Syndr.

[14] Nigatu T (2012). Integration of HIV and noncommunicable diseases in health care delivery in low- and middle-income countries. Prev Chronic Dis.

[15] Siu AL, US Preventive Services Task Force (2015). Screening for high blood pressure in adults: U.S. preventive services task Force recommendation statement. Ann Intern Med.

[16] World Health Organisation (2010). Package of essential noncommunicable (PEN) disease interventions for primary health Care in low-Resource Settings.

[17] World Health Organisation. Consolidated guidelines on the use of antiretroviral drugs for treating and preventing HIV infection: recommendations for a public health approach. Geneva: World Health Organisation; 2016. 27466667

[18] Uganda MoH (2016). Consolidated guidelines for prevention and treatment of HIV in Uganda.

[19] Chobanian AV, Bakris GL, Black HR, Cushman WC, Green LA, Izzo JL, Jones DW, Materson BJ, Oparil S, Wright JT, Roccella EJ, National Heart L, Blood Institute Joint National Committee on Prevention DE, Treatment of High Blood P, National High Blood Pressure Education Program Coordinating C (2003). The Seventh Report of the Joint National Committee on Prevention, Detection, Evaluation, and Treatment of High Blood Pressure: the JNC 7 report. JAMA.

[20] Lundgren JD, Battegay M, Behrens G, De Wit S, Guaraldi G, Katlama C, Martinez E, Nair D, Powderly WG, Reiss P, Sutinen J, Vigano A, Committee EE (2008). European AIDS clinical society (EACS) guidelines on the prevention and management of metabolic diseases in HIV. HIV Med.

[21] Whitworth JA, World Health Organization ISoHWG (2003). 2003 World Health Organization (WHO)/International Society of Hypertension (ISH) statement on management of hypertension. J Hypertens.

[22] Triant VA, Lee H, Hadigan C, Grinspoon SK (2007). Increased acute myocardial infarction rates and cardiovascular risk factors among patients with human immunodeficiency virus disease. J Clin Endocrinol Metab.

[23] Deeks SG, Phillips AN (2009). HIV infection, antiretroviral treatment, ageing, and non-AIDS related morbidity. BMJ..

[24] Semeere AS, Lwanga I, Sempa J, Parikh S, Nakasujja N, Cumming R, Kambugu A, Mayanja-Kizza H (2014). Mortality and immunological recovery among older adults on antiretroviral therapy at a large urban HIV clinic in Kampala, Uganda. J Acquir Immune Defic Syndr.

[25] Deeks SG (2009). Immune dysfunction, inflammation, and accelerated aging in patients on antiretroviral therapy. Top HIV Med.

[26] Hunt PW, Brenchley J, Sinclair E, McCune JM, Roland M, Page-Shafer K, Hsue P, Emu B, Krone M, Lampiris H, Douek D, Martin JN, Deeks SG (2008). Relationship between T cell activation and CD4+ T cell count in HIV-seropositive individuals with undetectable plasma HIV RNA levels in the absence of therapy. J Infect Dis.

[27] Bavinger C, Bendavid E, Niehaus K, Olshen RA, Olkin I, Sundaram V, Wein N, Holodniy M, Hou N, Owens DK, Desai M (2013). Risk of cardiovascular disease from antiretroviral therapy for HIV: a systematic review. PLoS One.

[28] D'Ascenzo F, Cerrato E, Biondi-Zoccai G, Moretti C, Omede P, Sciuto F, Bollati M, Modena MG, Gaita F, Sheiban I (2012). Acute coronary syndromes in human immunodeficiency virus patients: a meta-analysis investigating adverse event rates and the role of antiretroviral therapy. Eur Heart J.

[29] Rhew DC, Bernal M, Aguilar D, Iloeje U, Goetz MB (2003). Association between protease inhibitor use and increased cardiovascular risk in patients infected with human immunodeficiency virus: a systematic review. Clin Infect Dis.

[30] World Health Organization (2021). Updated recommendations on service delivery for the treatment and care of people living with HIV.

[31] Drain PK, Hong T, Hajat A, Krows M, Govere S, Thulare H, Moosa MYS, Bassett I, Celum C (2019). Integrating hypertension screening at the time of voluntary HIV testing among adults in South Africa. PLoS One.

[32] Mitambo C, Khan S, Matanje-Mwagomba BL, Kachimanga C, Wroe E, Segula D, Amberbir A, Garone D, Malik PR, Gondwe A, Berman J (2017). Improving the screening and treatment of hypertension in people living with HIV: an evidence-based policy brief by Malawi's knowledge translation platform. Malawi Med J.

[33] Kwarisiima D, Atukunda M, Owaraganise A, Chamie G, Clark T, Kabami J, Jain V, Byonanebye D, Mwangwa F, Balzer LB, Charlebois E, Kamya MR, Petersen M, Havlir DV, Brown LB (2019). Hypertension control in integrated HIV and chronic disease clinics in Uganda in the SEARCH study. BMC Public Health.

[34] Rohwer A, Uwimana Nicol J, Toews I, Young T, Bavuma CM, Meerpohl J (2021). Effects of integrated models of care for diabetes and hypertension in low-income and middle-income countries: a systematic review and meta-analysis. BMJ Open.

[35] US Preventive Services Task Force (2021). Screening for hypertension in adults: US preventive services task Force reaffirmation recommendation statement. JAMA.

[36] Heller DJ, Balzer LB, Kazi D, Charlebois ED, Kwarisiima D, Mwangwa F, Jain V, Kotwani P, Chamie G, Cohen CR, Clark TD, Ayieko J, Byonanabye DM, Petersen M, Kamya MR, Havlir D, Kahn JG (2020). Hypertension testing and treatment in Uganda and Kenya through the SEARCH study: an implementation fidelity and outcome evaluation. PLoS One.

[37] Muddu M, Tusubira AK, Sharma SK, Akiteng AR, Ssinabulya I, Schwartz JI (2019). Integrated hypertension and HIV care cascades in an HIV treatment program in eastern Uganda: a retrospective cohort study. J Acquir Immune Defic Syndr.

[38] Ahmad K, Jafar TH (2005). Prevalence and determinants of blood pressure screening in Pakistan. J Hypertens.

[39] Egan BM, Zhao Y, Axon RN (2010). US trends in prevalence, awareness, treatment, and control of hypertension, 1988-2008. JAMA..

[40] Hajjar I, Kotchen TA (2003). Trends in prevalence, awareness, treatment, and control of hypertension in the United States, 1988-2000. JAMA..

[41] Mills EJ, Beyrer C, Birungi J, Dybul MR (2012). Engaging men in prevention and care for HIV/AIDS in Africa. PLoS Med.

[42] Cornell M, Myer L (2013). Men and mortality: sex inequality in health outcomes. AIDS..

[43] Cornell M, Cox V, Wilkinson L (2015). Public health blindness towards men in HIV programmes in Africa. Tropical Med Int Health.

[44] Mozaffarian D, Benjamin EJ, Go AS, Arnett DK, Blaha MJ, Cushman M, de Ferranti S, Despres JP, Fullerton HJ, Howard VJ, Huffman MD, Judd SE, Kissela BM, Lackland DT, Lichtman JH, Lisabeth LD, Liu S, Mackey RH, Matchar DB, McGuire DK, Mohler ER, Moy CS, Muntner P, Mussolino ME, Nasir K, Neumar RW, Nichol G, Palaniappan L, Pandey DK, Reeves MJ, Rodriguez CJ, Sorlie PD, Stein J, Towfighi A, Turan TN, Virani SS, Willey JZ, Woo D, Yeh RW, Turner MB, American Heart Association Statistics C, Stroke Statistics S (2015). Heart disease and stroke statistics--2015 update: a report from the American Heart Association. Circulation..

[45] Prevention CfDCa (2016). Integrating HIV and hypertension management to improve lives in Malawi.

[46] International Epidemiologic Databases to Evaluate AIDS. https://www.iedea.org/. Accessed 12 Dec 2021.

